# Rare variants in *FANCA* induce premature ovarian insufficiency

**DOI:** 10.1007/s00439-019-02059-9

**Published:** 2019-09-18

**Authors:** Xi Yang, Xiaojin Zhang, Jiao Jiao, Feng Zhang, Yuncheng Pan, Qiqi Wang, Qing Chen, Baozhu Cai, Shuyan Tang, Zixue Zhou, Siyuan Chen, Hao Yin, Wei Fu, Yang Luo, Da Li, Guoqing Li, Lingyue Shang, Jialing Yang, Li Jin, Qinghua Shi, Yanhua Wu

**Affiliations:** 1 Obstetrics and Gynecology Hospital, NHC Key Laboratory of Reproduction Regulation (Shanghai Institute of Planned Parenthood Research), State Key Laboratory of Genetic, Engineering at School of Life Sciences, Fudan University, Shanghai, 200011 China; 2 Shanghai Key Laboratory of Female Reproductive Endocrine Related Diseases, Shanghai, 200011 China; 3 Center of Reproductive Medicine, Shengjing Hospital of China Medical University, Shenyang, 110004 China; 4 The Research Center for Medical Genomics, Key Laboratory of Cell Biology, Ministry of Public Health, Key Laboratory of Medical Cell Biology, Ministry of Education, College of Basic Medical Science, China Medical University, Shenyang, 110001 China; 5 The First Affiliated Hospital of USTC, The CAS Key Laboratory of Innate Immunity and Chronic Diseases, School of Life Sciences, CAS Center for Excellence in Molecular Cell Science, University of Science and Technology of China, Hefei, 230027 China; 6 Shanghai Ji Ai Genetic and IVF Institute, Fudan University, Shanghai, 200011 China; 7 National Demonstration Center for Experimental Biology Education, School of Life Sciences, Fudan University, Shanghai, 200433 China

## Abstract

Premature ovarian insufficiency (POI) is a major cause of reduced female fertility and affects approximately 1% women under 40 years of age. Recent advances emphasize the genetic heterogeneity of POI. Fanconi anemia (FA) genes, traditionally known for their essential roles in DNA repair and cytogenetic instability, have been demonstrated to be involved in meiosis and germ cell development. Here, we conducted whole-exome sequencing (WES) in 50 Han Chinese female patients with POI. Rare missense variants were identified in *FANCA* (*Fanconi anemia complementation group A*): c.1772G > A (p.R591Q) and c.3887A > G (p.E1296G). Both variants are heterozygous in the patients and very rare in the human population. In vitro functional studies further demonstrated that these two missense variants of *FANCA* exhibited reduced protein expression levels compared with the wild type, suggesting the partial loss of function. Moreover, mono-ubiquitination levels of FANCD2 upon mitomycin C stimulation were significantly reduced in cells overexpressing *FANCA* variants. Furthermore, a loss-of-function mutation of *Fanca* was generated in C57BL/6 mice for in vivo functional assay. Consistently, heterozygous mutated female mice (*Fanca*^**+*/*−**^) showed reduced fertility and declined numbers of follicles with aging when compared with the wild-type female mice. Collectively, our results suggest that heterozygous pathogenic variants in *FANCA* are implicated in non-syndromic POI in Han Chinese women, provide new insights into the molecular mechanisms of POI and highlight the contribution of *FANCA* variants in female subfertility.

**Electronic supplementary material:**

The online version of this article (10.1007/s00439-019-02059-9) contains supplementary material, which is available to authorized users.

## Introduction

Premature ovarian insufficiency (POI) is characterized by amenorrhea for more than 4 months and twice serum FSH > 25 IU/L (interval of more than 1 month) before 40 years of age (Webber et al. 2016). POI usually leads to female subfertility and affects approximately 1% women under 40 years (Coulam et al. 1986). Genetic etiologies account for approximately 20–25% of POI patients, but recent advances emphasize genetic heterogeneity (Jiao et al. 2015). POI causative genes isolated in POI pedigrees are mainly enriched in DNA damage repair, homologous recombination and meiosis (Jiao et al. 2018), including *CSB*-*PGBD3* (Qin et al. 2015), *MCM8* (Bouali et al. 2017; Tenenbaum-Rakover et al. 2015), *MCM9* (Fauchereau et al. 2016; Wood-Trageser Michelle et al. 2014), *MSH4* (Carlosama et al. 2017), *MSH5* (Guo et al. 2017) and *STAG3* (Caburet et al. 2014; Colombo et al. 2017; Le Quesne et al. 2016). Nevertheless, the genetic architecture underlying sporadic POI remains complicated since either gene variants or inheritance patterns are quite different among individuals.

Fanconi anemia (FA) is a rare autosomal recessive disease diagnosed through progressive bone marrow failure (BMF) at childhood along with high incidence of cancer susceptibility (Ceccaldi et al. 2016). During the past decades, 22 FA genes with essential roles in DNA interstrand cross-link (ICL) repair have been cloned (Deans and West 2011; Knipscheer et al. 2009). *FANCA* is most frequently mutated in FA, as bi-allelic mutations of *FANCA* accounting for 60‒70% of the cases (Neveling et al. 2009). Generally, the FA pathway is activated by ICL damage during the S phase. FANCA protein is one member of the FA core complex with ubiquitin E3 ligase activity, which induces the mono-ubiquitination of the FANCI/FANCD2 complex. DNA damage is further repaired by downstream proteins via homologous recombination (HR) mechanism to ensure the genome stability (Ceccaldi et al. 2016). Despite ICL repair, recent advances have emphasized the involvement of FA genes in fertility maintenance. Approximately half of female FA patients were reported to be infertile (Alter et al. 1991), and the mice with FA gene deficiency showed female subfertility of different degrees (Tsui and Crismani 2019). Moreover, the essential roles of FA genes in double-strand break (DSB) repair during HR in meiosis have been characterized (Tsui and Crismani 2019). For example, BRCA2 (FANCD1) is required for localization of RAD51 and DMC to DSBs to initiate DSB repair in meiosis, and persistent DSBs were observed in *Brca2*-mutated mice (Jensen et al. 2010; Sharan et al. 2004). However, the mutation patterns of FA genes contributing to human infertility remain largely unknown.

To explore the candidate pathogenic genes of POI, whole-exome sequencing (WES) was conducted in 56 Han Chinese women with POI from two centers in China. Two heterozygous rare missense variants in *FANCA*, c.1772G > A (p.R591Q) and c.3887A > G (p.E1296G), were identified in two sporadic POI cases. Further in vitro functional analysis demonstrated that both were partially loss-of-function missense variants. A heterozygous loss-of-function model for mouse ortholog *Fanca* was utilized for in vivo functional assays, and the female mice showed remarkable subfertility and impaired follicle development. Our experimental observations in humans and mice strongly suggest that heterozygous pathogenic *FANCA* variants can induce sporadic POI.

## Materials and methods

### Study participants, WES and data processing

56 Han Chinese women with POI were included in this study. The inclusion criteria consisted of primary or secondary amenorrhea for at least 4 months before 40 years of age, along with two measurements of abnormal serum FSH levels (> 25 IU/L). All the subjects with POI in this study had a normal 46,XX karyotype. Women with ovarian surgery or radiotherapeutic or chemotherapeutic interventions were excluded. The processing of WES and data analysis were as previously described (Wang et al. 2019). Primers for amplification and Sanger sequencing of the variants identified by WES are shown in Table S1.

### Plasmid construction and mutagenesis

The human full-length *FANCA* cDNA was synthesized (Shanghai Generay Biotech) and constructed into the pCMV-Myc vector (Takara). Site-directed mutagenesis was performed to generate two missense variants (R591Q, E1296G) of *FANCA* following the standard procedures of KOD-Plus-Mutagenesis kit (Toyobo). The plasmids were verified by Sanger sequencing before functional studies. The relevant primers are shown in Table S2.

### Cell culture and transfection

U2OS cells were purchased from the Cell Bank of the Chinese Academy of Sciences (Shanghai, China). U2OS cells were cultured in DMEM (Gibco) supplemented with 10% FBS (Gibco) and 1% penicillin–streptomycin–neomycin (PSN) antibiotic mixture (Gibco) at 37 °C with 5% CO_2_. To evaluate the transfection efficiency, *FANCA* plasmids were co-transfected with pEGFP-N2 vector (Clontech) into U2OS cells using Lipofectamine 3000 (Invitrogen) according to the manufacturer’s instructions. In experiments evaluating cell sensitivity to ICL damage, 2 μM mitomycin C (MMC; Sigma) was added into the culture medium 24 h after transfection.

### Western blotting

The experimental details were as previously described (Dou et al. 2019). The related antibodies included anti-FANCA (1:500 dilution, Abcam), anti-FANCD2 (1:2000 dilution, Abcam), anti-GFP (1:5000 dilution, Sigma), HRP labeled anti-β-actin (1:10,000, Proteintech Group) and HRP labeled goat anti-mouse/rabbit IgG (1:3000, DingGuo Bio).

### Mouse model

All the animal studies were performed in C57BL/6 mice. The heterozygous *Fanca* loss-of-function mice (*Fanca*^+*/−*^) were generated using CRISPR-Cas9 technology at University of Science and Technology of China. Primers for mice genotype identification are shown in Table S3.

### Mouse ovarian follicle counting

Ovary samples were fixed in 4% paraformaldehyde overnight, embedded in paraffin and then sectioned into 5 μm thickness. One in every five sections was stained with hematoxylin and eosin (H&E) and counted. Only follicles with clearly visible nucleus and normal morphology were counted. Follicle classification was determined by Pedersen’s system (Pedersen 1970). The results are reported as the average number of follicles counted in one ovary per female mouse.

### Statistical analysis

Comparisons of quantitative data were performed by Student’s *t* test. *P* < 0.05 was considered to be significantly different, and *P* < 0.01 was considered to be extremely significantly different. * represents *P* < 0.05, ** represents *P* < 0.01, and *** represents *P* < 0.001.

## Results

### Heterozygous rare variants of *FANCA* in sporadic cases with POI

We performed genetic analysis in 56 Han Chinese women with POI. To unravel the potential pathogenic variants in these cases, WES analysis was performed as previously described (Wang et al. 2019). As shown in Fig. S1, the data quality of variant calling and number of reads > 50 were assessed before genetic analysis. Then, genetic variants in the exonic and splicing regions were chosen, and the variants with minor allele frequencies > 0.001 according to the 1000 Genomes Project (1 KG) and ExAC Browser were excluded. Nonsynonymous variants predicted to be deleterious by all five bioinformatics tools including SIFT (Kumar et al. 2009), PolyPhen-2 (Adzhubei et al. 2010), MutationTaster (Schwarz et al. 2014), CADD (Kircher et al. 2014) and DANN (Quang et al. 2014) were taken as candidate pathogenic variants. In this study, the variants located in the novel genes, which are functionally related to ovary development but have not been reported as non-syndromic POI causative genes (Jiao et al. 2015), were preferred.

To our interest, two heterozygous missense variants in *FANCA* were identified in two unrelated non-syndromic POI subjects. Subject F027 carrying *FANCA* c.1772G > A (p.R591Q) variant had normal puberty and was menopausal at the age of 24. Subject L010 carrying *FANCA* c.3887A > G (p.E1296G) variant had primary amenorrhea. Moreover, no other rare variant in known POI causative or candidate genes was observed in these two cases.

**Fig. 1:**
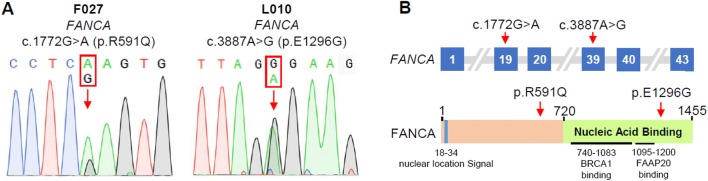
Identification of rare variants of *FANCA* in two patients with POI. **a** Heterozygous rare *FANCA* variants. The red arrows indicate the variant positions. **b** Schematic representations of the *FANCA* gene and FANCA protein. The red arrows indicate the variant positions

The two *FANCA* variants were confirmed by Sanger sequencing (Fig. 1a). The *FANCA* c.1772G > A variant has an extremely low frequency in human population, and is highly evolutionarily conserved according to Phastcons and Phylop scores (Table 1) (Pollard et al. 2010). The *FANCA* c.3887A > G variant is a novel missense variant, which changes a residue located in the nucleic acid binding domain of FANCA (Table 1; Fig. 1b). Notably, these two *FANCA* variants are also classified to be likely pathogenic following the American College of Medical Genetics (ACMG) guidelines (Table 1) (Richards et al. 2015). These observations suggest that these two heterozygous missense variants in *FANCA* are possibly pathogenic.

**Table 1:** Overview of the rare *FANCA* variants observed in Chinese patients with POI

Subject	cDNA change^a^	Protein change	ACMG category	Minor allele frequency^b^			Conservation^c^		Functional prediction^d^				
				1 KG	ExAC	gnomAD	Phastcons	Phylop	SIFT	PolyPhen-2	MutationTaster	CADD	DANN
F027	c.1772G > A	p.R591Q	LP	0	0.00016	0.00014	0.988	4.419	Damaging	Possibly damaging	Disease causing	5.172	0.999
L010	c.3887A > G	p.E1296G	LP	0	0	0	0.104	2.351	Damaging	Probably damaging	Disease causing	4.157	0.998

^a^The GenBank accession number of *FANCA* is NM_000135

^b^Minor allele frequencies were estimated according to the databases of the 1000 Genomes (1 KG) Project, ExAC and gnomAD

^c^The more conserved the position, the closer is the Phastcons score to 1. Positive score represents predicted conserved site by Phylop, while larger value means higher conservation

^d^Mutation assessment by the SIFT, PolyPhen-2, MutationTaster, CADD and DANN tools. Higher CADD and DANN scores suggest that variants are more likely to have deleterious effects. CADD cutoff is usually set as 4, while 0.93 is for the DANN cutoff

### *FANCA* variants reduced FANCA expression and impaired FANCD2 mono-ubiquitination in vitro

**Fig. 2:**
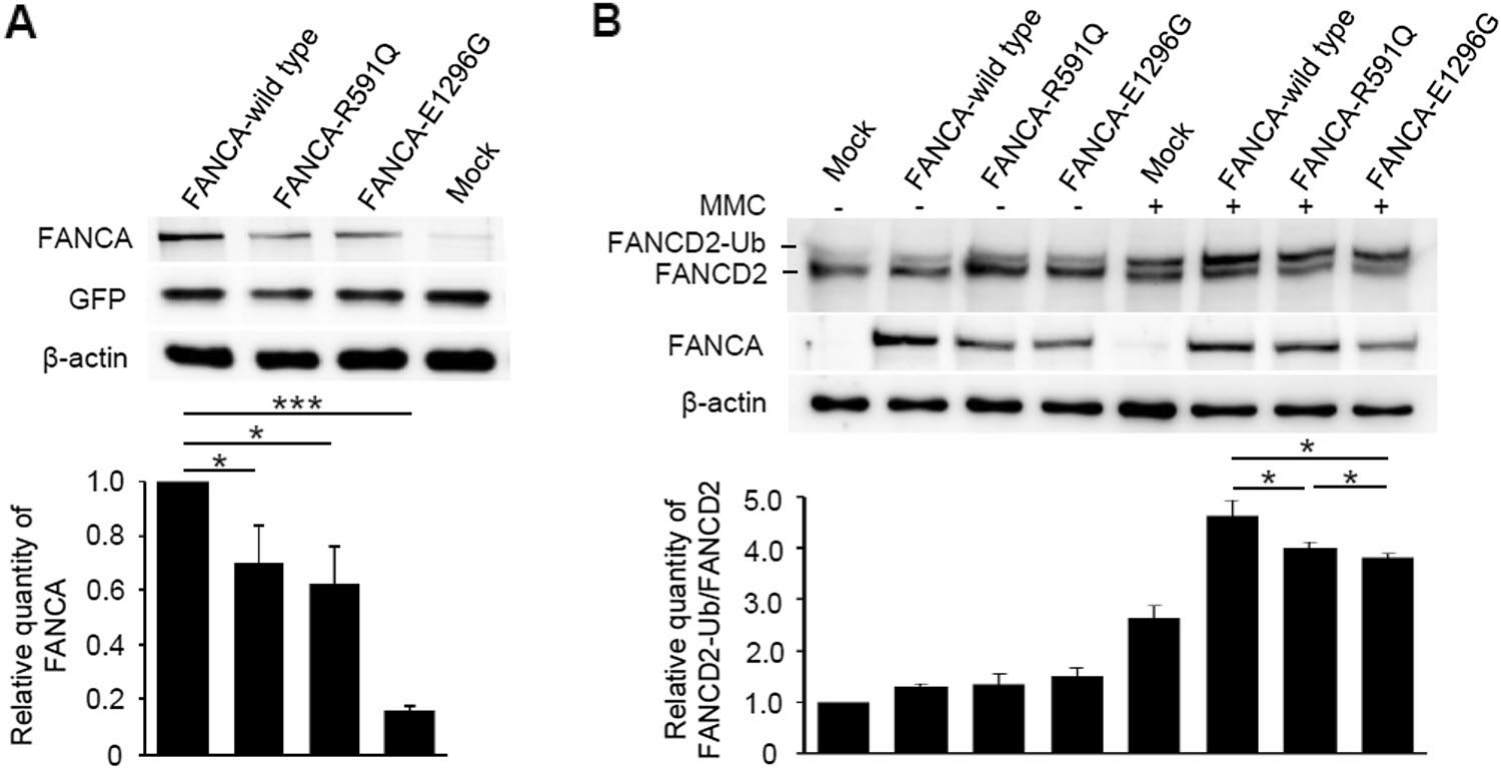
Reduced expression levels and activities of the rare *FANCA* variants. **a** Western blot analysis of the protein expression levels of wild-type FANCA and two altered proteins (R591Q and E1296G). An equal amount of indicated *FANCA* expression plasmids was co-transfected with pEGFP-N2 into U2OS cells. The densitometric units of altered FANCA proteins were normalized to that of the wild-type FANCA. Values are expressed as mean ± SD, *N* = 4. GFP was used to evaluate the transfection efficiency, and β-actin was used as a loading control. **b** Western blot analysis of mono-ubiquitinated FANCD2 in U2OS cells transfected with indicated *FANCA* expression plasmids with or without MMC (2 μM) treatment for 24 h. The densitometric units show the ratios of mono-ubiquitinated FANCD2 to unubiquitinated FANCD2, normalized to that of cells transfected with empty vector without MMC treatment. Values are expressed as mean ± SD, *N* = 4. β-Actin was used as a loading control. MMC, mitomycin C. *P < 0.05; **P < 0.01; ***P < 0.001

In vitro functional assays were performed to further investigate the biological effects of the two *FANCA* variants. The putative effects on *FANCA* protein expression were first investigated in the over-expression assays. U2OS cells were transfected with equal amounts of recombinant plasmids of wild-type and mutated *FANCA*, respectively. Western blot analysis revealed that the amounts of R591Q and E1296G altered proteins were nearly half of the wild-type FANCA (Fig. 2a), indicating that these two variants may affect protein expression levels. Furthermore, mono-ubiquitination levels of FANCD2 under DNA damage were investigated. In the absence of MMC, FANCD2 mono-ubiquitination levels were relatively low, and there was no obvious difference between wild-type and mutated FANCA. Upon MMC treatment, while the wild-type FANCA significantly increased FANCD2 mono-ubiquitination levels, both R591Q and E1296G altered proteins exhibited statistically reduced effects on FANCD2 mono-ubiquitination (Fig. 2b). These results suggest that under some environmental pressure, such as MMC treatment, FANCA altered proteins may be partially loss of function. Remarkably, E1296G altered protein showed severer effects on FANCD2 mono-ubiquitination than R591Q, which was consistent with the severity of clinical phenotypes in the affected cases.

### *Fanca*^+*/*−^ female mice showed decreased fertility and impaired follicle development

To investigate the in vivo effect of *FANCA* on ovarian function, a mouse model carrying a heterozygous loss-of-function mutation (c.3585insA; p.E1195Efs*6) of *Fanca* in C57BL/6 background was utilized (Fig. S2a, S2b). Both mRNA and protein expression levels of *Fanca* in the ovary of the mutated mice were almost half of the wild-type control, and no Fanca truncate protein was detected in mutated mice (Fig. S2c, S2d).

**Fig. 3:**
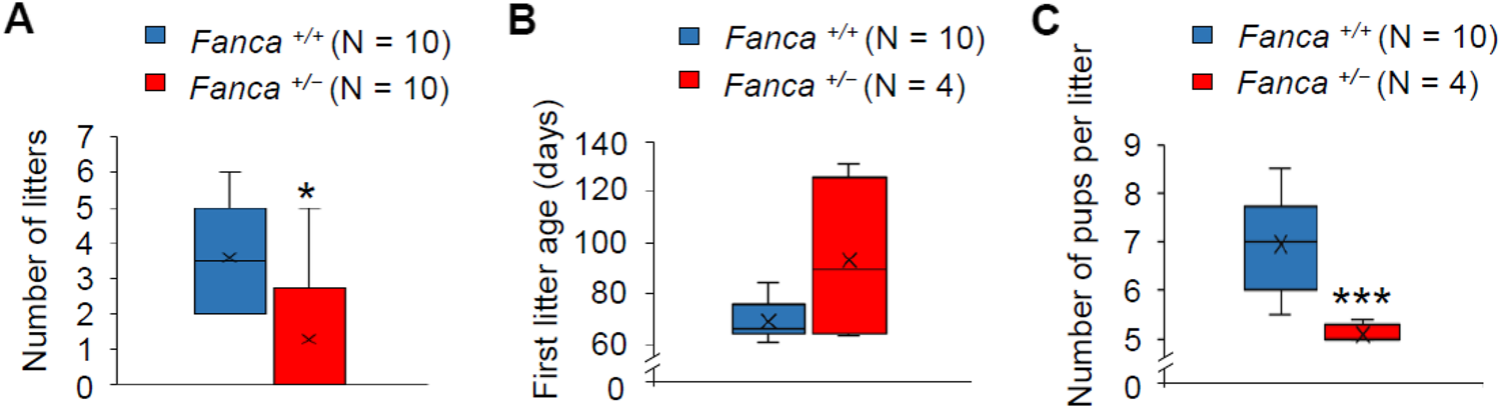
*Fanca*^+*/−*^ female mice manifested decreased fertility. **a** Number of litter sizes, **b** first litter ages, and **c** number of pups per litter in wild-type and *Fanca*^+*/−*^ female mice. Each female mouse mated with wild-type male mouse, respectively, from sexual maturity (6 weeks) to 6 months. “×” represents the average, horizontal lines represent the median, upper and lower edges of the box represent the up and down four digits, and the upper and lower bars represent the maximum and minimum values. No outlier was detected

The breeding assays were performed by mating *Fanca*^+*/*−^ female mice with wild-type males from sexual maturity to 6 months. By comparison with the wild-type female mice whose average number of litters was 3.6, *Fanca*^+*/*−^ female mice exhibited obvious subfertility with phenotypic variation among individuals: 6 mice never gave a birth in the mating period, whereas the other 4 mice littered 1–5 times, respectively (Fig. 3a). The first litter age of the *Fanca*^+*/*−^ females which had given births was much later than that of the wild type (Fig. 3b). Consistently, the number of pups per litter was reduced in *Fanca*^+*/*−^ females (Fig. 3c).

**Fig. 4:**
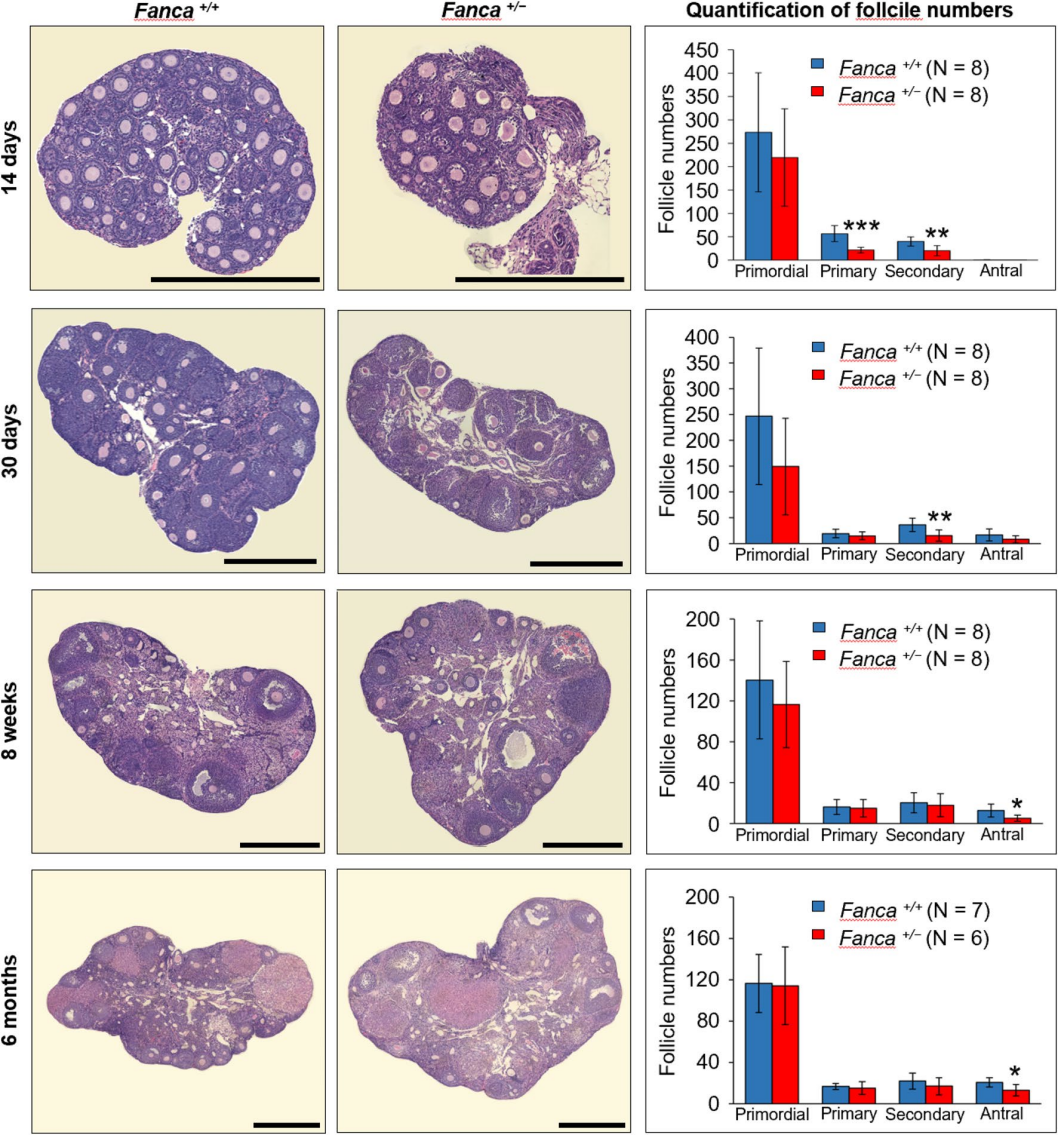
The numbers of follicles decreased in the ovaries of *Fanca*^+*/−*^ female mice. Representative images of H&E staining of mouse ovaries at ages of 14 days, 30 days, 8 weeks and 6 months. Scale bars represent 500 μm. The numbers of primordial, primary, secondary and antral follicles were counted and expressed as mean ± SD. *P < 0.05; **P < 0.01; ***P < 0.001

To investigate whether the subfertility of *Fanca*^+*/*−^ female mice was due to ovarian dysfunction, anatomy analysis of female reproductive system was performed. There was no apparent difference in morphology and size of the uteruses and ovaries between *Fanca*^+*/*−^ and wild-type mice (Fig. S3). H&E staining also indicated that the morphology of ovaries in *Fanca*^+*/*−^ mice was normal, and all types of follicles could be found (Fig. 4, left panel). However, follicle counting revealed a decreasing pattern of follicle numbers with aging (Fig. 4, right panel). The number of primordial follicles in *Fanca*^+*/*−^ mice decreased by approximately 20‒40% compared to that in wild-type mice before sexual maturity (14 days and 30 days). At the same time, the numbers of both primary and secondary follicles in the ovaries of *Fanca*^+*/*−^ mice were significantly less than that in wild type. After sexual maturity (8 weeks), the number of antral follicles in the ovaries of *Fanca*^+*/*−^ mice reduced to nearly half of that of the wild type. This decreased pattern of antral follicles in *Fanca*^+*/*−^ mice continued till 6 months. Meanwhile, massive atretic follicles appeared in the ovaries of *Fanca*^+*/*−^ mice by comparison with the wild type (Fig. S4). Altogether, the histological analysis strongly suggests that partial loss of function of *Fanca* in mice impaired normal follicle development, leading to partial ovarian dysfunction and subfertility, which resembles POI phenotype in humans.

## Discussion

The reduced fertility in approximately half FA female patients strongly suggests the contribution of the FA pathway in human fertility. Ovary is one of the organs that is most susceptible to unrepaired DNA damage induced by either genetic variations or environmental factors (Oktem and Oktay 2007). More recently, emerging evidence has shown that some pathogenic variants in several FA genes lead to human infertility without development of FA phenotypes. Different compound heterozygous *BRCA2* mutations have been recently identified to cause ovary dysgenesis or non-syndromic POI (Qin et al. 2019; Weinberg-Shukron et al. 2018). Similarly, different bi-allelic mutations in *FANCM* were suspected in independent studies as being the causes for early menopause (Catucci et al. 2018), non-syndromic POI (Fouquet et al. 2017), and non-obstructive azoospermia (Kasak et al. 2018; Yin et al. 2019). Moreover, the genome-wide association studies (GWAS) in European ancestry populations suggest that *FANCC* is a high risk locus of polycystic ovary syndrome (Hayes et al. 2015), and *FANCI* is implicated in age at menopause (Stolk et al. 2012).

A variety of bi-allelic pathogenic variants in *FANCA* have been identified in FA patients, several of which manifested POI as part of their phenotypes (Kimble et al. 2018). Furthermore, a nonsynonymous variant rs2239359 in the *FANCA* gene was found to be associated with POI in Korean women through GWAS study (Pyun et al. 2014). Herein, we first reported two pathogenic *FANCA* variants in non-syndromic POI patients through WES analysis. To our interest, the novel missense mutation of *FANCA* (c.3887A > G) was identified in patient L010 with primary amenorrhea and another rare missense mutation (c.1772G > A) was in the second amenorrhea case F027. Consistent results were gained in the functional study: the *FANCA* c.3887A > G variant exhibited a severer effect on FANCA expression and activity than the c.1772G > A variant. All these findings suggest a genotype–phenotype correlation for *FANCA* pathogenic variants in female fertility.

On the other hand, independent studies have indicated that different inheritance patterns of DNA repair genes can lead to phenotypic variability. Heterozygous mutations of *BRCA2*, *BRIP1* (*FANCJ*) and *PALB2* (*FANCN*) are not sufficient to develop FA phenotypes, but they are associated with a dramatic increased risk for breast, ovarian and other cancers (Berwick et al. 2007; Catucci et al. 2014; Seal et al. 2006). Bi-allelic mutations in *ERCC6* (excision repair cross-complementing 6), a family member of *ERCC4* (*FANCQ*), lead to Cockayne syndrome (CS, OMIM #133540) characterized by severe growth and developmental retardation, progressive neurological dysfunction and symptoms of premature aging (Falik-Zaccai et al. 2008), while heterozygous pathogenic variants result in non-syndromic POI (Qin et al. 2015). Our present study indicated *FANCA* as another good example of DNA repair gene with genetic pleiotropy. Further mechanistic studies elucidating the contribution of each *FANCA* variant to ovarian functions are awaited to provide important evidence for accurate diagnosis in clinic. The phenotypes of sporadic POI are highly variable in clinic, probably due to different environmental factors, genetic background as well as different pathogenic variants.

Additional evidence demonstrating the relationship between FA genes and fertility comes from homozygous recessive mouse models of FA genes. Most of these mice show severely reduced female fertility accompanied by different phenotypes, such as smaller ovary sizes, reduced numbers of germ cells and follicles, decreased litter sizes or even complete sterility (Tsui and Crismani 2019). Among those, two types of *Fanca* knockout mice were constructed by gene targeting approaches and exhibited declined female fertility at different degrees. The first *Fanca*-deficient strain with a deletion of exons 4‒7 was constructed in 129Ola, C57BL/6 and FVB mixed strain, showing severe infertility before 20 weeks and almost no follicles in ovaries (Cheng et al. 2000). Another *Fanca*-deficient strain with a deletion of exons 1‒6 was constructed in two mouse strains, and it was found that the loss of *Fanca* exhibited reduced litter sizes in 129S6 strain, but infertility with almost all PGCs lost in fetal ovaries in C57BL/6 strain (Wong et al. 2003).

These observations from different models and groups prompt our attention to the fact that the fertility phenotypes in different mice strains carrying different *Fanca* mutants are variable. It might partially explain the fact that no obvious differences between heterozygous *Fanca*-null and wild-type mice was observed in previous two *Fanca* mutants, but we found that heterozygous loss-of-function mutation of *Fanca* in C57BL/6 mice could affect female fertility. Furthermore, a truncated *Fanca* mRNA transcript was detected in the *Fanca*-deficient mice (Wong et al. 2003), so it is reasonable to doubt that a partial Fanca protein might be existing to partially complement wild-type *Fanca* function. To further confirm our finding in *Fanca*^+*/*−^ mice, we thoroughly recorded follicle development in mice of different ages. Histological analysis indicates that a remarkable increasing number of the follicles in the ovaries of *Fanca*^+*/*−^ mice could not develop normally or further undergo atresia by comparison with that in wild-type mice, which explains the decreased female fertility in *Fanca*^+*/*−^ mice. Taken together, we raised the hypothesis that the differences in *Fanca* mutation and genetic background of mouse strains might contribute to the phenotypic variations in mouse models, and the ovary function might be correlated to the expression levels of *Fanca* and its altered transcripts.

The diverse roles of FA genes in DSB repair during HR have been discovered more recently (Alavattam et al. 2016). FANCA is responsible for the nucleus localization of the FA core complex (Garcia-Higuera et al. 2000) and promotes DNA DSB repair by catalyzing single-strand annealing and strand exchange (Benitez et al. 2018). Fancd2 failed to accumulate on meiotic chromosomes in *Fanca* knockout cells (Alavattam et al. 2016), indicating that the function of FANCA in meiosis is related to the activation of FANCD2. In our study, we also observed that two *FANCA* variants found in POI patients exhibited reduced activities in regulating mono-ubiquitination of FANCD2, which may affect the DNA damage repair efficiency and correctness of oocytes in the process of meiosis. The “memory” of meiotic defects before the diplotene arrest may guide oocytes with unrepaired DNA damage undergoing apoptotic pathways during follicular development (Qiao et al. 2018), which is consistent with our observations of decreased numbers of follicles in each developmental stages and boomed numbers of atresia follicles with aging in heterozygous *Fanca*-mutated mice. In addition, an accelerated ovary aging related to the decline in follicle number in women carrying *BRCA1* (*FANCS*) and *BRCA2* germline mutations has been recently reported (Lin et al. 2017).

In summary, our findings in human subjects and mice suggest that heterozygous pathogenic variants in *FANCA* could affect female fertility, providing novel insights into the molecular diagnosis of female subfertility and genetic counseling for women who are at a risk for POI.

### Web resources

The URLs for the data presented herein are as follows:

1000 Genomes Project, http://browser.1000genomes.org

CADD, http://cadd.gs.washington.edu

DANN, http://cbcl.ics.uci.edu/public_data/DANN

ExAC Browser, http://exac.broadinstitute.org

GenBank, http://www.ncbi.nlm.nih.gov/genbank

gnomAD browser, http://gnomad.broadinstitute.org

MutationTaster, http://www.mutationtaster.org

OMIM, http://www.omim.org

PolyPhen-2, http://genetics.bwh.harvard.edu/pph2/

SIFT, http://sift.bii.a-star.edu.sg

UCSC Genome Browser, http://genome.ucsc.edu.

## Electronic supplementary material

Below is the link to the electronic supplementary material.

Supplementary material 1 (DOCX 1516 kb)
