# Supplementary material for: Rare variants in FANCA induce premature ovarian insufficiency
Source: Hum Genet. 2019 Sep 18;138(11):1227–36. doi: 10.1007/s00439-019-02059-9 (PMC6874525; doi:10.1007/s00439-019-02059-9)
Supplement: Supplementary file 1 — Supplementary material 1 (DOCX 1516 kb) [file 439_2019_2059_MOESM1_ESM.docx]

Original Article, *Human genetics*

**Rare variants in *FANCA* induce premature ovarian insufficiency**

Xi Yang *et al*.

**Supplementary meterials**


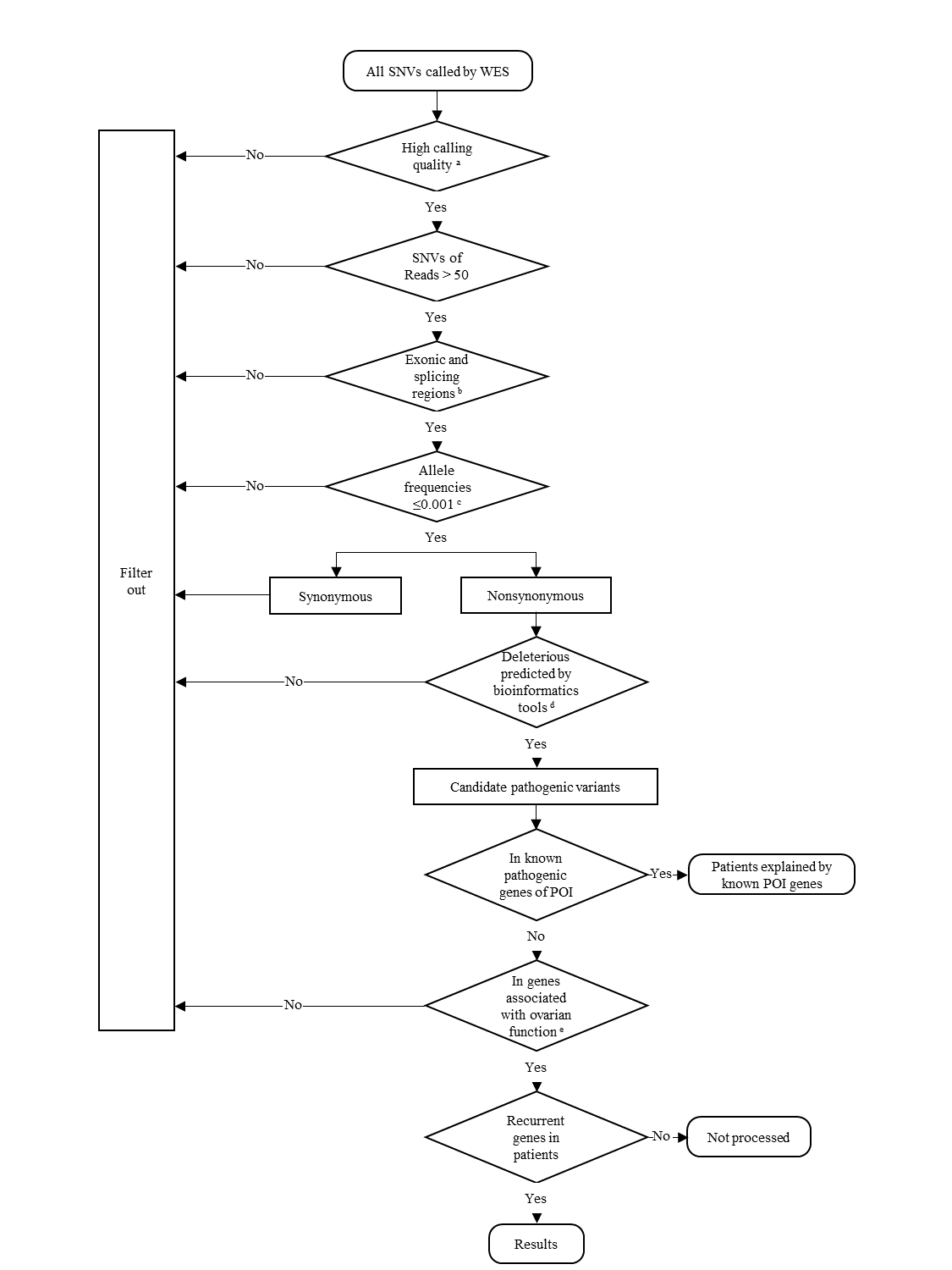


**Figure S1 Workflow of WES data filtering.** ^a^ SNVs were called using GATK and varscan. ^b^ Splicing region includes a range of 15 bp upstream and downstream of the exons. ^c^ Allele frequencies of SNVs were < 0.001 in the 1KG Project, ExAC and gnomAD databases. ^d^ SNVs were predicted to be deleterious by the SIFT, PolyPhen-2, MutationTaster, CADD and DANN tools. ^e^ Information from the gene ontology annotation of biological process.


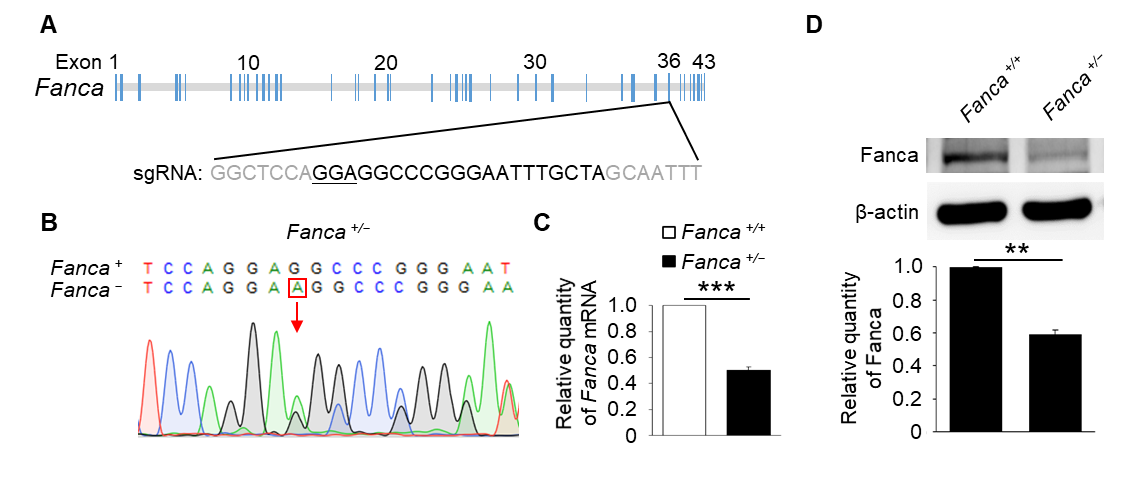


**Figure S2 Construction of the *Fanca^+/−^* mouse model.** A) Schematic representation of the sgRNA targeting the exon 36 of mouse *Fanca* using CRISPR-Cas9 technology. The gDNA sequence is in gray, the sgRNA sequence is in black, and the PAM motif is underlined. B) Sanger sequencing of *Fanca****^+/−^*** mice. The red arrow indicates the position of an insertion mutation, leading to frame shift of *Fanca*. C) Quantitative real time PCR analysis of *Fanca* mRNA expression levels in mouse ovaries. Values are expressed as mean ± SD, N = 3. D) Western blot analysis of Fanca protein expression levels in mouse ovaries. The densitometric units of Fanca protein in *Fanca****^+/−^*** mouse ovary was normalized to that of wild type controls. β-actin was used as a loading control. Values are expressed as mean ± SD, N = 3. **, *P* < 0.01; ***, *P* < 0.001.


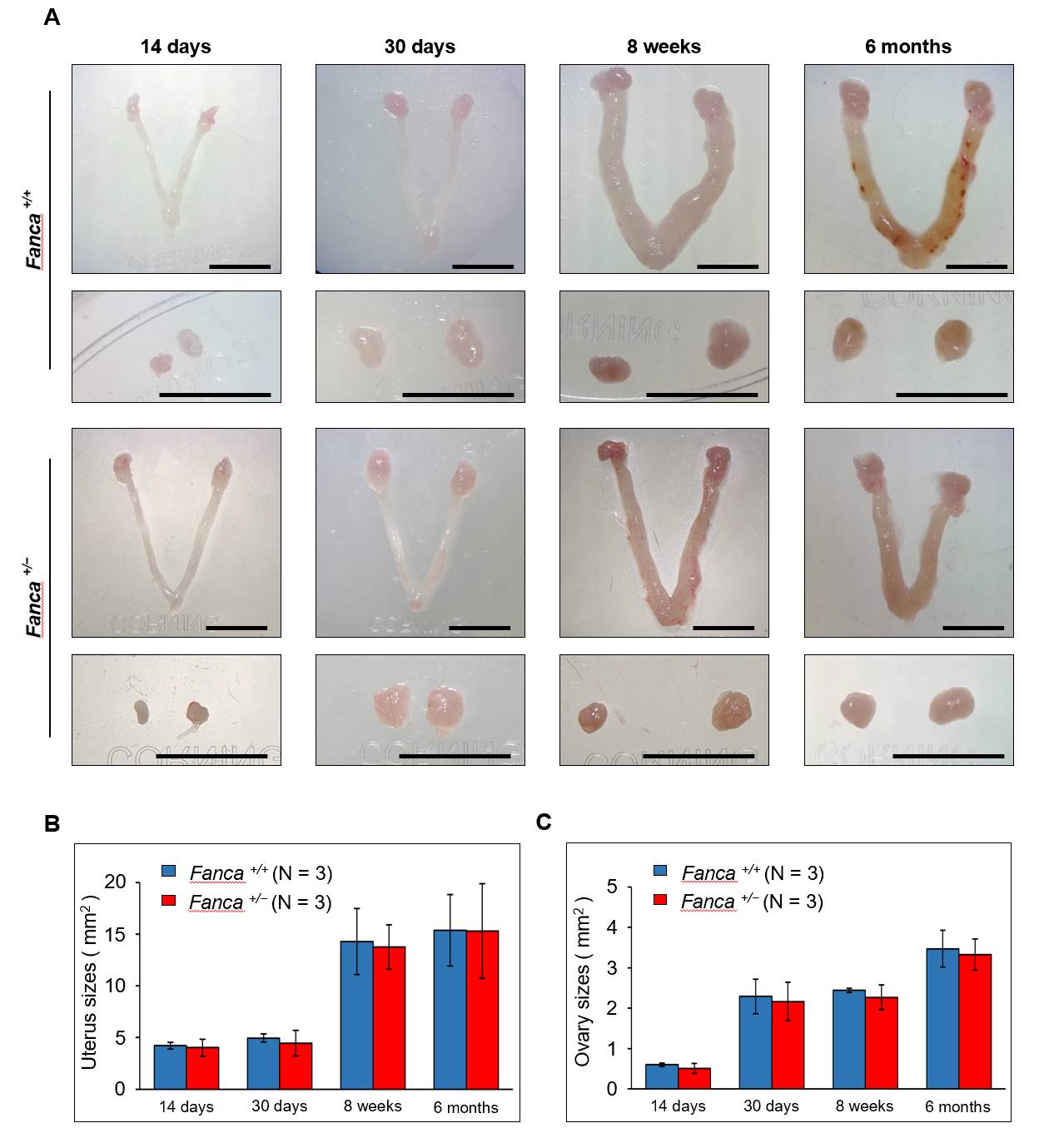


**Figure S3 Morphology of the reproductive system in *Fanca^+/−^* female mice.** A) Uteruses (upper panel) and ovaries (bottom panel) of both wild type and *Fanca****^+/−^*** mice were dissected at 14 days, 30 days, 8 weeks and 6 months, respectively. After that, these samples were washed in sterile PBS, put in petri dishes and photographed under an anatomic microscope. Scale bars represent 5 mm. B) Uterus sizes were measured by the product of the uterus length and the diameter of the mid-stage uterus. Values are expressed as mean ± SD, N = 3. C) Ovary sizes were measured by the product of long and short ovary diameters. Values are expressed as mean ± SD, N = 3.


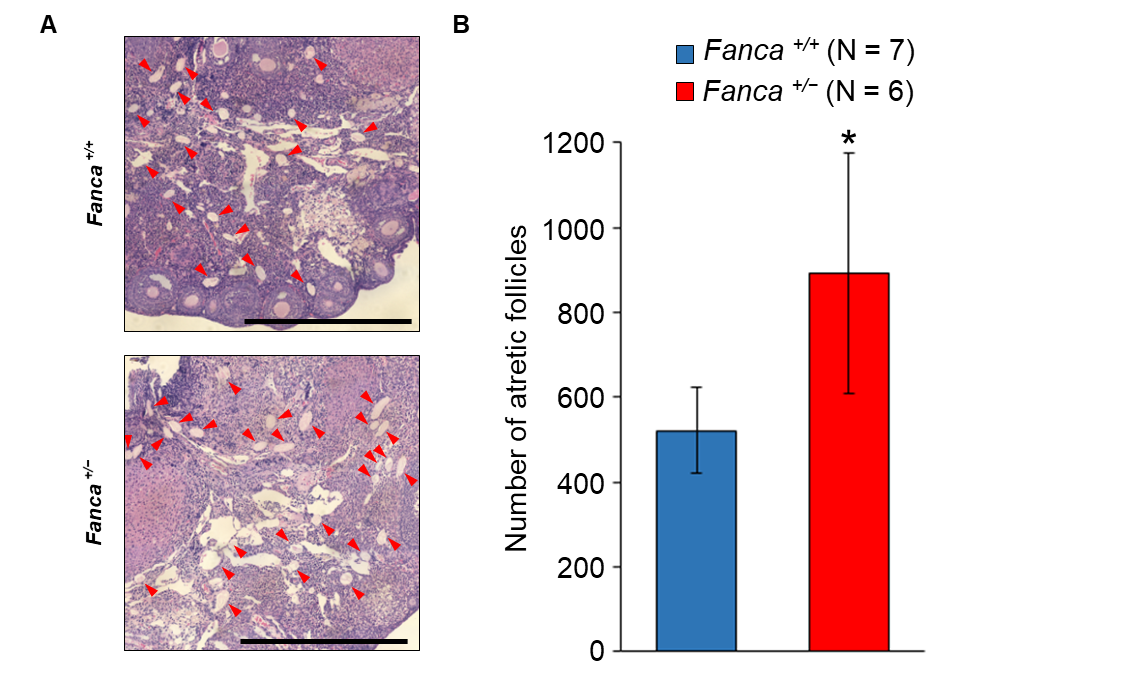


**Figure S4 Quantitative analysis of atretic follicles in wild type and *Fanca^+/−^* mice at age of 6 months.** A) Representative images of H&E staining of mouse ovaries at age of 6 months. Arrows indicate atretic follicles. Scale bars represent 500 μm. B) Statistical analysis of the numbers of atretic follicles. Values are expressed as mean ± SD. *, *P* < 0.05.

**Table S1** Primers for amplification and Sanger sequencing of the variants identified by WES.

| **Primer Name** | **Primer Sequence** |
| --- | --- |
| F027-F | 5'-GTCAGGGACTTTGGGGA-3' |
| F027-R | 5'-GCATCGTGGCTTTGGA-3' |
| L010-F | 5'-GTTGACCAGTGAGCCAGT-3' |
| L010-R | 5'-TGAGCCTCTTCTGTCCAG-3' |

**Table S2** Primers for construction of recombinant plasmids carrying *FANCA* variants.

| **Primers for site-directed mutagenesis** | |
| --- | --- |
| R591Q-KOD-F | 5'-AAGTGCTCCCCAAAGTCCCTGACT-3' |
| R591Q-KOD-R | 5'-GAGGTGTGAGCAGGGCG-3' |
| E1296G-KOD-F | 5'-GGAAGAGGAAGATATCCTGGCTGG-3' |
| E1296G-KOD-R | 5'-CTAAACACTCGAGGAT-3' |
| **Primers for Sanger sequencing** | |
| R591Q-Sanger-F | 5'-AGTACCGCTCCCTCCTC-3' |
| R591Q-Sanger-R | 5'-TAACATCACGCTGGCTG-3' |
| E1296G-Sanger-F | 5'-CTCTCCCCTGAGGCTG-3' |
| E1296G-Sanger-R | 5'-TGTGCTTGTATCCCCAG-3' |

**Table S3** Primers for mouse genotyping.

| **Primer Name** | **Primer Sequence** |
| --- | --- |
| *Fanca*-F | 5'-GACCTATGAATTCTAGCCCAG-3' |
| *Fanca*-R | 5'-CCTGATGAAACCAGTCTTCAG-3' |
